# DNA Methylation Biomarkers Predict Progression-Free and Overall Survival of Metastatic Renal Cell Cancer (mRCC) Treated with Antiangiogenic Therapies

**DOI:** 10.1371/journal.pone.0091440

**Published:** 2014-03-14

**Authors:** Inga Peters, Natalia Dubrowinskaja, Mahmoud Abbas, Christoph Seidel, Michael Kogosov, Ralph Scherer, Kai Gebauer, Axel S. Merseburger, Markus A. Kuczyk, Viktor Grünwald, Jürgen Serth

**Affiliations:** 1 Department of Urology and Urologic Oncology, Hannover Medical School, Hannover, Germany; 2 Department of Pathology, Hannover Medical School, Hannover, Germany; 3 Department of Oncology/Hematology/Bone MarrowTransplantation/Pneumology, University Medical Center Eppendorf, Hamburg, Germany; 4 Department of Biometry, Hannover Medical School, Hannover, Germany; 5 Clinic for Hematology, Hemostasis, Oncology and Stem Cell Transplantation, Hannover Medical School, Germany; Nanjing Medical University, China

## Abstract

VEGF-targeted therapy increases both the progression-free (PFS) and overall survival (OS) of patients with metastasized renal cell cancer (mRCC). Identification of molecular phenotypes of RCC could improve risk-stratification and the prediction of the clinical disease course. We investigated whether gene-specific DNA hypermethylation can predict PFS and OS among patients undergoing anti-VEGF-based therapy. Primary tumor tissues from 18 patients receiving targeted therapy were examined retrospectively using quantitative methylation-specific PCR analysis of *CST6*, *LAD1*, hsa-*miR*-124-3, and hsa-*miR-*9-1 CpG islands. PFS and OS were analyzed for first-line and sequential antiangiogenic therapies using the log rank statistics. Sensitivity and specificity were determined for predicting first-line therapy failure. Hypermethylation of *CST6* and *LAD1* was associated with both a shortened PFS (log rank p = 0.009 and p = 0.004) and OS (p = 0.011 and p = 0.043). The median PFS observed for the high and low methylation groups of *CST6* and *LAD1* was 2.0 vs.11.4 months. *LAD1* methylation had a specificity of 1.0 (95% CI 0.65–1.0) and a sensitivity of 0.73 (95% CI 0.43–0.90) for the prediction of first-line therapy. *CST6* and *LAD1* methylation are candidate epigenetic biomarkers showing unprecedented association with PFS and OS as well as specificity for the prediction of the response to therapy. DNA methylation markers should be considered for the prospective evaluation of larger patient cohorts in future studies.

## Introduction

Renal cell cancer (RCC) is one of the top ten causes of cancer deaths in industrial countries [1]. Though recent improvements in targeted therapy have resulted in prolonged survival of patients with metastatic RCC (mRCC), the overall outcome is still poor [2], [3].

Due to different available compounds and a growing number of new agents affecting molecularly targeted structures, such as vascular endothelial growth factor (VEGF) and mammalian target of rapamycin (mTOR) signaling [4] an optimal sequence of targeted therapies might exist for patients, potentially increasing survival with mRCC treatment. Although prognostic models, such as the MSKCC (Memorial Sloan Kettering Cancer Center) and Heng scoring systems, have been reported to be independent predictors of clinical outcome, [5], [6] discrimination between outcomes is still limited. Tumor-specific biologically based parameters have been suggested to improve these issues [7].

Most RCCs have clear cell (ccRCC) histology and exhibit functional inactivation of the von Hippel-Lindau (*VHL*) gene due to mutations or epigenetic silencing in approximately 80% of tumors [8], [9]. However, the progression-free (PFS) and overall survival (OS) of patients with mRCC are independent of the loss of *VHL* function [10]. In contrast, blood-based analysis of single nucleotide polymorphisms (SNPs) potentially affecting sunitinib target genes and ligands identified two polymorphisms in *VEGFR3* that are associated with the PFS, but not OS, of patients undergoing targeted therapy [11]. Measurement of serum carbonic anhydrase IX (CA9) protein levels in metastatic ccRCC patients revealed significantly decreased OS among patients with higher CA9 serum concentrations [12]. An individual advantage of tissue- or blood-based measurements for patients undergoing therapy is perceptible, but the accuracy, sensitivity, and specificity of these methods have not yet been reported or validated [13].

Although large patient cohorts have been subjected to exom-wide mutational analyses, only a limited number of genes other than *VHL* and polybromo 1 (*PBRM1*) have been identified to have mutations in RCC, and most with low frequency [14]. Therefore, the limited number of frequently mutated genes reduces the probability of identifying mutation-based predictors with appropriate sensitivity and specificity. DNA methylation of CpG islands (CGIs) in a substantial number of regulatory or tumor suppressor genes has been identified as a functional surrogate of mutations and has been reported to be a frequent event in RCC [15]. Moreover, functional loss of *VHL* has been found to associate with broadened appearance of epigenetic alterations [16], and all of the second-frequent mutations are related to altered chromatin/histone stabilization or modification, mechanisms linked to CGI methylation and gene expression [17]. Therefore, the frequent detection of epigenetic alterations in RCC differentiates RCC tumor biology and provides candidates for novel diagnostic, prognostic, or predictive markers [18]. CGI methylation in several genes has already been identified as candidate prognosticators independent from clinicopathological parameters [19]–[21]. However, epigenetic biomarkers predicting the clinical course of mRCC patients subjected to targeted therapy, have, to the best of our knowledge, not been reported.

We hypothesized that CGI methylation is related to the response to therapy, as well as the survival of mRCC patients undergoing antiangiogenic therapy. We investigated four candidate genes, three of which, cystatin E/M (*CST6*) and the micro RNA genes *miR-9-1* and *miR-124-3,* were identified recently with tumor-specific CGI hypermethylation and a possible association with the prognosis of RCC patients [19], [21], [22]. The Ladinin 1 (*LAD1*) gene was identified recently by our group as a new candidate methylation marker in RCC showing univariate association with adverse clinicopathological parameters such as tumor grade, lymph node metastasis, status of distinct metastasis and advanced disease (unpublished data).

*LAD1* encodes an anchoring filament protein, a component of the basement membrane that likely contributes to the stability of the epithelial-mesenchymal interaction [23].

The present study investigated whether a DNA methylation mark can predict the response of targeted antiangiogenic therapy of mRCC patients and describes the identification of two DNA methylation markers in the *CST6* and *LAD1* CGIs as candidate epigenetic predictors of the PFS and OS of mRCC patients undergoing targeted therapy.

## Materials and Methods

### Ethics Statement

Informed consent was obtained from each patient, and the local ethics committee (Ethic Committee; Prof. H. D. Tröger, Hannover Medical School, Carl-Neuberg-Str. 1, Hannover, Germany; Study_No: 1213–2011) specifically approved this study. Patients agreed in a written form for utilization of tissue specimen for basic research. A written statement of our ethic committee for this study was obtained.

### Patient Characteristics and Treatment Regimens

Clinicopathological data, corresponding tissues, and follow-up data including the PFS and OS of patients with mRCC who were treated with first-line VEGF-targeted therapy were collected between November 2005 and October 2011 in the Clinic of Hematology and the Department of Urology and Urologic Oncology at Hannover Medical School (Table 1). The MSKCC score or ECOG performance status were not available. Patients received the following treatment regimens in the first-line setting: sunitinib (n = 12, 67%), sorafenib (n = 4, 22%), axitinib (n = 1, 5.5%), and bevacizumab (n = 1, 5.5%).

**Table 1:** Patient characteristics.

Patient No.	Age (years)	Sex	RCC type	iTNM status	First-line treatment	PFS (months)	OS (months)	*Response
1	68	F	clear cell	T1bNxMx	Sunitinib	1.34	2.47	NE
2	66	M	clear cell	T4N1Mx	Sunitinib	1.77	1.77	PD
3	57	M	clear cell	T3bNxMx	Sunitinib	2.76	3.62	PD
4	59	M	chromophobe	T4N2Mx	Sunitinib	1.70	9.76	PD
5	72	F	clear cell	T3bN0Mx	Sorafenib	5.52	23.64	SD
6	48	F	clear cell	T3NxMx	Sunitinib	2.26	2.99	PD
7	62	M	clear cell	T2aNxMx	Sunitinib	2.63	3.25	PD
8	80	F	clear cell	n.a.	Sunitinib	0.88	26.14	SD
9	50	M	clear cell	T2NxMx	Sorafenib	11.86	13.68	SD
10	71	F	clear cell	T1bN1M1	Axitinib	13.70	19.04	SD
11	69	F	clear cell	T1bNxMx	Bevacizumab	6.21	11.07	SD
12	57	M	clear cell	T3N0Mx	Sunitinib	11.50	29.77	SD
13	49	F	clear cell	T3aNxM1	Sunitinib	11.27	26.86	PR
14	60	M	clear cell	T3bNxMx	Sunitinib	0.42	0.76	NE
15	54	M	clear cell	T2aNxMx	Sorafenib	3.03	13.05	PD
16	53	M	clear cell	T1NxMx	Sorafenib	43.66	59.28	SD
17	64	M	clear cell	n.a.	Sunitinib	30.31	59.24	CR
18	51	M	papillary	T1aNxMx	Sunitinib	1.08	3.42	PD

Note:

*Response: according to RECIST 1.1 criteria.

Sex: Male, Female.

NE: not evaluable du e to RECIST 1.1 criteria.

CR: complete response.

PR: partial response.

SD: stable disease.

PD: progressive disease.

Age: At the beginning of first-line therapy.

n.a: not available.

iTNM: initial TNM status of primary RCC.

PFS was defined as the time from the beginning of the first day of systemic therapy to the detection of a progressive event according to RECIST 1.1 criteria on a computer tomography (CT) scan [24]. OS was defined as the period from the first day of systemic therapy until the patient’s death or censored at the last follow-up. The terminus “not evaluable” in Table 1 describes patients with a PFS <2 months due to an early cessation of therapy caused by toxicity or early death before the first recommended CT scan after therapy was initiated. The initial TNM classification of primary tumors was evaluated according to the Union for International Cancer Control 2002 classification [25]. Patient follow-up included up to three sequence changes in the therapy regimen.

The terms ´prognostic` and ´predictivè were used according to the definition by the National Cancer Institute [26].

### Tissue Specimens, Isolation, and Bisulfite Conversion of Tumor DNA

Independent control of histopathology, tumor cell content of routine pathological specimens, and the selection of tissue areas for tissue extraction were determined by the pathologist. Subsequently, cylinders 1.5 mm in length and 2 mm in height were stamped out from the formalin-fixed and paraffin-embedded (FFPE) tissue blocks using an 18 Charrière core stamp and subjected to DNA isolation. Genomic DNA was extracted using the automated MagNA Pure LC 2.0 system and MagNA Pure LC DNA isolation kit II - tissue (Roche Diagnostics Deutschland, Roche Applied Science, Mannheim, Germany). The quality of extracted DNA was assessed using spectrophotometry, gel electrophoresis, and quantitative PCR, which characterized the yield, purity, and grade of degradation of isolated DNA. Bisulfite conversion of DNA was carried out using the EZ DNA Methylation-Gold Kit (Zymo Research Corporation, Irvine CA, USA) and 1 µg of isolated DNA. Fully methylated and converted DNA, as well as unmethylated bisulfite-converted DNA controls, were used as reported previously [21].

### Quantitative Methylation-specific Real-time PCR Analysis

Quantitative real-time fluorimetric 5′ exonuclease PCR (qMSP) assays were performed to quantify the CGI methylation levels of *CST6, LAD1,* hsa*-miR-124-3,* and hsa-*miR-9-1*. The methylation analysis of hsa*-miR-124-3* was carried out as described previously [21]. qMSP systems were established for *CST6, LAD1,* and hsa-*miR-9-1* using Beacon Designer software (PREMIER Biosoft, Palo Alto CA, USA). The base positions of investigated CGI sites for *CST6, LAD1,* hsa*-miR-124-3,* and hsa-*miR-9-1* are presented in Table 2. The base positions refer to the USCS Genome Browser [27]. The qMSP systems were characterized as described for the hsa*-miR-124-3* methylation measurements [21]. Duplicate real-time PCRs were performed on an ABI 7900HT (Life technologies, Foster City, USA) in 384-well plates as described previously [21]. Experimenters were blinded to the histopathological and clinical status of the samples. Relative methylation levels were calculated as an analogue of the ΔΔCt method by normalizing the difference in CGI methylation determined by real-time detection and independent internal control measurements to the corresponding difference in the fully methylated DNA control samples as described previously [21], [28].

**Table 2:** Gene informations.

	*CST6*	*LAD1*	*miR-9-1*	*miR-124-3*
Chromosome	11q13	1q32.1	1q22	20q13.33
Name	Cystatin E/M	Ladinin 1	micro RNA9-1	micro RNA124-3
GeneID	1474	3898	407046	406909
*CpG Island*				
# number of CpG sites	59	54	99	424
# base position (bp)	65779312–65777967	201368561–201369032	156390404–156391581	61806255–61810867
bp of CpG sitesinvestigated byqMSP	65779535, ∼541, ∼600, ∼604,∼612, ∼620, ∼630, ∼640,∼644, ∼647	201368651, ∼669, ∼672, ∼689,∼693, ∼696, ∼700, ∼704, ∼713,∼725, ∼733	156390684, ∼701, ∼745,∼747, ∼753, ∼758,∼764, ∼783	61809002, ∼007, ∼026,∼035, ∼044, ∼059,∼065, ∼072

Note: Gene informations according to the USCS Genome Browser [27].

### Statistical Analysis of Survival

Kaplan-Meier plots were used to present relative survival in the PFS and OS analyses following dichotomization of tumors into high and low methylation phenotypes. The median survival and corresponding 95% confidence intervals (CIs) were reported. Differences in PFS and OS were tested using log-rank statistics and median survival ratios calculated. *P*-values <0.05 were considered significant. To allow a comparison with the literature, univariate Cox regression analyses were performed to estimate hazard ratios (HRs). To calculate sensitivity and specificity for therapy failure, a PFS cutoff value of 6 months was used for dichotomization [29] into therapy responders and non-responders.

The heat map and receiver operating characteristic curves were constructed using the heatmap2 and ROCR function in the R package (version 2.11.0.1) with a default clustering algorithm and gplot package [30].

## Results

### Bimodal Distribution of Relative Methylation Levels in the CGIs of Candidate Genes

**Figure 1:**
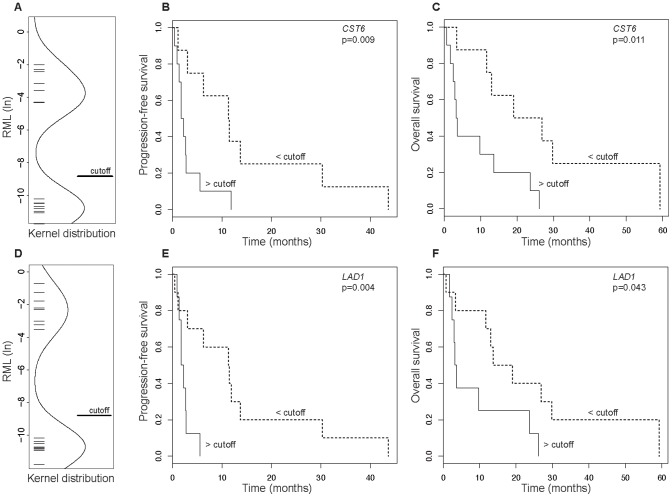
Survival analyses. A and D. istribution of the relative methylation values of *CST6* (A) and *LAD1* (D) in mRCC patients. A cutoff value is presented for dichotomization. B and E. Kaplan-Meier plots of the progression-free survival of mRCC patients dichotomized by high and low methylation of *CST6* (B) and *LAD1* (E). C and F Kaplan-Meier plots of the overall survival of mRCC patients dichotomized by high and low methylation of *CST6* (C) and *LAD1* (F).

Quantitative methylation analyses of the CGIs of *CST6*, *LAD1*, hsa-*miR-124-3,* and hsa-*miR-9-1* revealed the presence of a bimodal distribution of relative methylation values (Figure 1A and D; data not shown for hsa-*miR-124-3* and hsa-*miR-9-1*). Applying a single cutoff value of 0.02% (corresponding to −8.75 in the ln-scale used for Kernel density plots in Figure 1A and D) for relative methylation, high and low methylated epigenotypes were uniformly distinguished for all of the analyzed genes and used for consistent dichotomization in survival analyses.

### Analysis of PFS

Kaplan-Meier and log rank analysis of PFS in high and low methylated tumors demonstrated a significant difference for both *CST6* and *LAD1*. High methylation was associated with a median survival of 2.0 months, compared to 11.4 months among patients with low methylation (p = 0.009 and p = 0.004, Table 3A). In contrast, neither *miR-124-3* nor *miR-9-1* demonstrated a statistical relationship with PFS (p = 0.339 and p = 0.319).

**Table 3:** Survival analyses.

A)	PFS	Median survival (months, 95% CI)		Median survival ratio (high/low)	
	p-value*	low methylation	high methylation		HR (95% CI)**
*CST6*	**0.009**	11.4 (6.2–NE)	2.0 (1.3–NE)	0.175	4.1 (1.3–12.6)
*LAD1*	**0.004**	11.4 (3.0–NE)	2.0 (1.7–NE)	0.175	6.4 (1.6–26.0)
*miR-124-3*	0.339	11.9 (6.2–NE)	2.6 (1.7–11.5)	0.218	1.8 (0.5–6.6)
*miR-9-1*	0.319	4.6 (1.3–NE)	2.7 (1.8–NE)	0.587	1.7 (0.6–4.7)
**B)**	**OS**	**Median survival (months, 95% CI)**		**Median survival ratio (high/low)**	
	**p-value** *	**low methylation**	**high methylation**		**HR (95% CI)** **
*CST6*	**0.011**	22.9 (13.1–NE)	3.4 (2.5–NE)	0.148	4.1 (13.0–13.4)
*LAD1*	**0.043**	16.4 (11.7–NE)	3.4 (3.0–NE)	0.207	2.9 (1.0–8.6)
*miR-124-3*	0.786	13.7 (11.7–NE)	9.8 (3.2–29.8)	0.715	0.8 (0.2–3.1)
*miR-9-1*	0.624	12.4 (3.4–NE)	14.4 (3.2–NE)	1.161	1.3 (0.5–3.6)

Abbreviations:

PFS: Progression-free survival.

OS: Overall survival.

NE: not estimable.

HR: Hazard ratio.

CI: Confidence interval.

*: log-rank statistical analysis.

**: Univariate Cox regression for purpose of comparision.

low methylation cutoff <8.75.

high methylation cutoff ≥8.75.

### Analysis of OS

Kaplan-Meier analysis and log rank statistics revealed that high methylation of *CST6* and *LAD1* was associated with impaired OS. A median OS of 22.9 and 3.4 months (p = 0.011, Table 3B) was obtained for low and high *CST6* methylation, respectively. A median OS of 16.4 and 3.4 months (p = 0.043, Table 3B) was obtained for low and high *LAD1* methylation.

### Analysis of Sensitivity and Specificity

**Figure 2:**
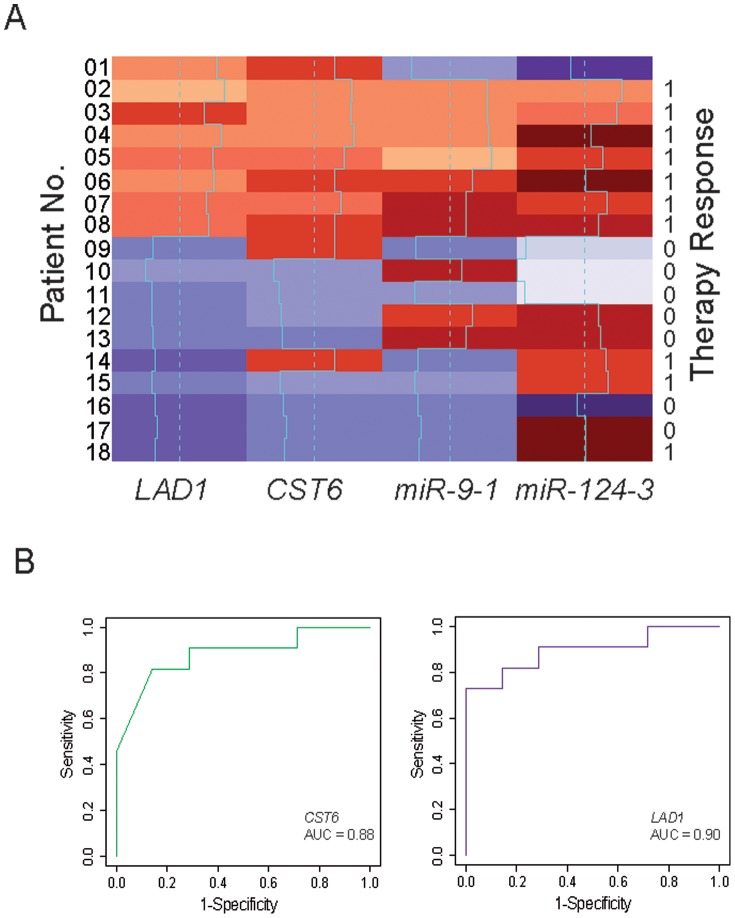
Heat map illustration of therapy response and Receiver Operating Characteristic Curves for *CST6* and *LAD1.* A. Heat map of normalized relative methylation values (natural logarithm) detected in *LAD1*, *CST6*, *miR-9-1,* and *miR-124-3* CGIs for each patient. Low to high methylation values are encoded as violet (low) to red (high) hues. The dashed and solid lines describe the median and individual methylation values, respectively. Patient numbers given on the left correspond to the numbering presented in Table 1. Therapy response (0) and therapy failure (1) are indicated for each patient on the right. Notably, all of the patients (no. 1–8) exhibiting high methylation of *LAD1* and 9 of 10 patients (no. 1–9, 14) exhibiting high methylation (red colored) of *CST6* were part of the non-responder (1) group. B. The receiver operating characteristics (ROC) curves illustrated the discrimination of methylation measurements and the area under the curve (AUC) shows that even with our small patient cohort, a robust result for the accuracy of both methylation markers (AUC *CST6* = 0.88 and AUC *LAD1* = 0.90) can be detected. The sensitivity (true positive rate) is plotted against 1-specificity (false positive rate).

To determine the sensitivity and specificity of *CST6* and *LAD1* methylation analyses for predicting first-line therapy failure, methylation values were dichotomized into low and high methylation phenotypes using the same cutoff value of 0.02% as specified above. PFS values were dichotomized using a cutoff of 6 months, a parameter that was previously suggested to better distinguish between therapy responders and non-responders [29]. High methylation of *LAD1* and *CST6* was a characteristic of failed therapy (Figure 2A). In the case of *LAD1,* all eight patients with high methylation were non-responders. The specificity was 1.0 (95% CI 0.65–1.0) and sensitivity 0.73 (95% CI 0.43–0.90) for the detection of therapy failure using *LAD1* methylation (Table 4), whereas the specificity was 0.86 (95% CI 0.49–0.97) and sensitivity 0.82 (95% CI 0.52–0.95) for *CST6* methylation (Table 4).

**Table 4:** Sensitivity and specificity.

	Sensitivity	95% CI	Specificity	95% CI	p-value*
*CST6*	0.818	0.52–0.95	0.857	0.49–0.97	**<0.001**
*LAD1*	0.727	0.43–0.90	1.000	0.65–1.00	**0.004**
*miR-124-3*	1.000	0.74–1.00	0.429	0.16–0.75	0.339
*miR-9-1*	0.636	0.35–0.85	0.571	0.25–0.84	0.319

CI: Confidence interval.

*log-rank test.

## Discussion

The clinical outcomes of patients with mRCC have improved since VEGF-targeted therapies and mTOR inhibitors were made available [2], [3]. However, the stratification of patients using biomarkers could allow a better understanding of drug resistance and identify an optimized patient-specific sequence of antiangiogenic therapies, improving individual survival [7]. Moreover, the side effects of anti-VEGF-based regimens, such as diarrhea, rash, hand-foot syndrome, hypertension, and asthenia, which often severely impair quality of life during treatment can be minimized.

We found that DNA hypermethylation of *CST6* and *LAD1* in primary RCC tumor tissue is significantly associated with the PFS of patients receiving anti-VEGF-based medication as a first-line therapy and also the OS of patients sequentially treated with anti-VEGF targeted drugs and mTOR inhibitors in second- and third-line therapy. Our methylation markers predicted therapy failure with high specificity and good sensitivity.

To the best of our knowledge, these findings are unprecedented in several respects, as previous studies were not tissue based and either provided no significant association with therapy response [12] or reported only limited statistical power [11]. While serum measurements of CA9 levels revealed no significant differences between therapy responders and non-responders [12] the analysis of genetic variants, possibly interacting with the sunitinib pathway, identified two *VEGFR3* SNPs to be associated with therapy response and PFS, but not with OS [11]. This study shows that individual biological variables may affect the response to therapy. On the other hand, our methylation-based candidate predictors go beyond the measurement of gene variants in several important aspects. First, the potential *LAD1* and *CST6* DNA methylation-based markers were measured in tumor cells, which directly exhibit tumor characteristics that may represent drivers of resistance and biological aggressiveness. Hypermethylation of *CST6* and *LAD1* exhibited prognostic and predictive value in our study and is a putative biomarker for patient selection. Based on the clinical outcomes in our study, different therapeutic strategies for hypermethylated tumors will be required. After the network of epigenetic alterations and biological behaviors has been untangled, additional novel targets of therapeutic interventions may be identified.

Whether the difference in epigenetic tissue- and genetic blood-based measurements accounts for both epigenetic markers being related to the PFS and OS of mRCC patients is an interesting question. Gene variants were only associated with PFS, a surrogate endpoint for survival measurements in mRCC that has possible limitations [31]. To the best of our knowledge, a tissue-based molecular marker has not previously been associated with OS. From a statistical point of view, our epigenetic study delivered higher HRs in survival analyses and provided a more balanced classification into responders and non-responders than the study by Garcia-Donas et al. [11], and therefore together contributing to a higher power of this study. Considering that a much smaller patient cohort was available for our measurements, our findings indicate that a strong effect has possibly been identified. Moreover, bearing in mind that a growing number of agents can be used for the treatment of mRCC, future identification of an optimum therapy regimen could be facilitated by epigenetic markers that allow good separation of patients into responders and non-responders.

Interestingly, the methylation levels of all candidate markers clearly decayed into easily distinguishable high and low methylation groups, eliminating the need to arbitrarily define cutoff points for dichotomization. Therefore, virtually no overlap existed between the responders and non-responders in the present study. Thus our *LAD1* and *CST6* methylation analyses yielded high specificities of 1.0 and 0.86 for the detection of therapy failure, underlining the possible relevance of these markers in mRCC.

This study may also answer whether DNA methylation-based prognosticators represent appropriate predictors of disease. Both *miR-9-1* and *miR-124-3* [21], [22] failed as predictors because no association was found with the PFS or OS of patients undergoing therapy. This finding might be explained by the fact that mRCC patients generally face a poor prognosis, and many tumors exhibit high methylation for the *miR* genes as expected for candidate prognosticators.

The independence from clinical or laboratory parameters could not be determined in the present study because the low sample numbers prevented multivariate analysis. Correspondingly, the relevant questions whether markers could be combined to optimize the predictive power or whether markers exhibit redundant information can only be answered in future studies by use of enlarged study cohorts.

However, the HRs observed for clinical parameters for patient outcome were lower with limited accuracy/discriminatory power. Our results require confirmation in an independent validation study including the consideration of clinical scoring systems as confounders.

In conclusion, our study identified *LAD1* and *CST6* CGI methylation as two epigenetic markers that are associated with the PFS and OS of mRCC patients undergoing antiangiogenic therapy. We have also shown the potential to improve the molecular prediction of the response to therapy. Our results further stress the notion that epigenetically altered RCCs exist, and novel specific strategies may be required to treat patients with such tumors.
